# Development and professional validation of an App to support Oral Cancer Screening

**DOI:** 10.1590/0103-6440202204895

**Published:** 2022-12-05

**Authors:** Talita Jordânia Rocha do Rêgo, José Vitor Mota Lemos, Amanda Pinheiro Leitão Matos, Caio Ferreira Freire Caetano, Thinali Sousa Dantas, Fabrício Bitu Sousa, Edgar Marçal de Barros, Paulo Goberlânio de Barros Silva

**Affiliations:** 1 Centro Universitário Christus, Departamento de Odontologia, Fortaleza, Ceará, Brazil.; 2 Universidade Federal do Ceará. Fortaleza, Ceará, Brazil.

**Keywords:** Squamous Cell Carcinoma, Family Health Strategy, Early Diagnosis, Mobile Apps, Smartphone

## Abstract

The objective of this study was to develop and validate an App for identifying risk factors for oral cancer. To this end, we developed an App (OCS: Oral Cancer Screening) with predictors of Oral Cancer (OC) and algorithm assembly to estimate the risk of its development. Methodology: Simulated clinical cases were designed so that 40 professionals with expertise in oral diagnostics could validate the algorithm and test its usability (SUS: System Usability Score) and acceptability (TAM: Technology Acceptance Model). Cronbach's alpha coefficient, Friedman/Dunn tests, and Spearman correlation evaluated the SUS and TAM scales. ROC curve was plotted to estimate the cutoff point of the algorithm in suggesting a high risk for OCS of the simulated cases. Chi-square and Fisher's exact tests were additionally used (p<0.05, SPSS v20.0). Results: Professionals with expertise in oral diagnosis had usability of 84.63±10.66 and acceptability of 84.75±10.62, which correlated positively (p<0.001, r=0.647). Acting in clinical areas of dentistry (p=0.034) and history of performing OC risk factor orientation (p=0.048) increased acceptability while acting in higher education increased usability (p=0.011). The cutoff point suggested by the App after validation of the simulated clinical cases showed high sensitivity of 84.8% and lower specificity of 58.4%. Conclusion: The OCS was effective and with adequate sensitivity, usability, and acceptability and may contribute to the detection of early oral lesions.

## Resumo

O objetivo deste estudo foi desenvolver e validar um aplicativo para identificação de fatores de risco para câncer bucal. Para tanto, desenvolvemos um aplicativo (OCS: Oral Cancer Screening) com preditores de Câncer Oral (CO) e montagem de algoritmo para estimar o risco de seu desenvolvimento. Metodologia: Casos clínicos simulados foram projetados para que 40 profissionais com expertise em diagnóstico oral pudessem validar o algoritmo e testar sua usabilidade (SUS: System Usabilidade Score) e aceitabilidade (TAM: Technology Acceptance Model). O coeficiente alfa de Cronbach, os testes de Friedman/Dunn e a correlação de Spearman avaliaram as escalas SUS e TAM. A curva ROC foi traçada para estimar o ponto de corte do algoritmo ao sugerir um alto risco para OCS dos casos simulados. Os testes do qui-quadrado e exato de Fisher foram usados adicionalmente (p<0,05, SPSS v20.0). Resultados: Profissionais com expertise em diagnóstico oral tiveram usabilidade de 84,63±10,66 e aceitabilidade de 84,75±10,62, que se correlacionaram positivamente (p<0,001, r=0,647). Atuar em áreas clínicas da odontologia (p=0,034) e histórico de realização de CO orientação de fator de risco (p=0,048) aumentaram a aceitabilidade enquanto atuar no ensino superior aumentou a usabilidade (p=0,011). O ponto de corte sugerido pelo App após validação dos casos clínicos simulados apresentou alta sensibilidade de 84,8% e baixa especificidade de 58,4%. Conclusão: O OCS foi eficaz e com sensibilidade, usabilidade e aceitabilidade adequadas e pode contribuir para a detecção de lesões orais precoces.

## Introduction

Considered as the sixth most prevalent malignant neoplasm globally, oral cancer has an incidence of 3.90/100,000 inhabitants, with a mortality rate of 1.94/100,000. In 5 years, 50% mortality was directly related to the tumor. In Brazil, 11,180 new cases in men and 4,010 in women per year are estimated for the triennium 2020-2022. There is an estimated risk of 10.69 new cases per 100,000 men and 3.71 per 100,000 women, occupying the fifth and thirteenth most frequent positions among the types of cancers, respectively ^1^ ^,^ ^2^ ^,^ ^3^.

In accordance with the alarming data on the incidence of oral cancer, it is necessary to plan and train professionals to perform early diagnosis and prevention programs. Oral cancer prevention can be classified as primary, secondary, and tertiary. Early detection of oral cancer by screening among high-risk groups can change the prognosis of these lesions and significantly reduce healthcare costs and mortality rates ^4^ ^,^ ^5^.

A comprehensive literature review described that the factors that most positively affect the early diagnosis of oral cancer are cancer screening programs and early search promoted by patients and professionals involved ^6^. However, programs and strategies for an active search of lesions and screening programs require specific knowledge about the epidemiology of the analyzed region and constant training and qualification of the members involved, in Brazil, most often the community health agents (CHA) ^7^, which makes these processes considerably costly and locoregionalized ^8^. 

Amidst the development of mobile devices, several health services have been modernized, and even highly complex colorimetric assays have been analyzed on smartphones ^9^. In 2008, Roobol suggested a series of algorithms based on guidelines and regional epidemiological surveys to aid in prostate cancer risk prediction ^10^. After a few years, Pereira-Azevedo et al. ^11^ developed an iOS platform that assists in predicting the risk of developing prostate cancer.

Platforms have been developed to assist dentists in diagnosing potentially malignant and malignant lesions of the oral cavity ^12^. The use of these interactive technologies will soon constitute an important tool in reducing the existing sociodemographic barriers between the health professionals and the affected population, speeding up the diagnosis and treatment of these patients ^13^ ^,^ ^14^. Therefore, this study aimed to develop and professionally validate an *App* for the screening of oral cancer in the Family Health Strategy.

## Methods

### Ethical aspects of research

The project was submitted to Plataforma Brasil and presented to the Research Ethics Committee of the Centro Universitário Christus (Unichristus) with opinion number 2,327,073, as per the attached document.

### Prospecting and App development platform

An updated search was carried out between the *App*s on April 18, 2022 in the PubMed database and in the official store of the operating systems (Google Play for Android and the *App* Store for iOS). In PubMed, the keywords “e-Health” and “oral cancer” were used, 19 articles were located. Articles that developed, tested or analyzed *App*s or software that helped in the diagnosis or monitoring of cancer patients were considered, selecting 4 articles ^15^ ^,^ ^16^ ^,^ ^17^ ^,^ ^18^, but none of these articles had the same proposal as this study.

For mobile operating systems the following keywords were used “Oral cancer”, “Mouth cancer”, “Cancer bucal”, “Cancer oral” and “Cancer de boca”, after deleting the duplicate *App*s and performing an analysis, 11 *App*lications were selected (Bucal® ,Cancer Risk Calculator®, DoctOral®, ESMO Interactive Guidelines®, Estomato*App*®, Head & Neck Cancer Manager®, Head and Neck (Oral) Cancer Risk Assessment Tool®, Mouth Cancer®, Treatments - FAQ®, Oral Cancer Screening *App*®, RiskOCA® e Teeth4Life®) that addressed the topic of OC, but they focus on patient guidelines for self-examination of the mouth, guided diagnosis for oral lesions, self-care during cancer treatment, methods of preventing oral cancer. None of them had the objective of tracking risk factors as predictors by professionals of referring patients to primary health care ^19^.

Subsequently, the *App* OCS was developed by professionals in dentistry and computing in the Department of Technological Innovation of the Unichristus. It was established that the developed version would be for one of the leading existing mobile platforms, Android. 

### Development of a risk stratification algorithm for oral cancer

An alpha version of *App* was developed using odds ratios described by a case-control study of Andrade of risk factors for the development of oral cancer in a population in northeastern Brazil ^20^. To identify the odds ratios of each factor, we built a formula based on the product of the risk indices as suggested by Mendonça et al. ^21^ to generate a number that could estimate the risk of developing OC. This formula was constructed using numerical data obtained from studies of risk factors for OC. Numerical data retrieved from these articles were prevalence ratios of each risk factor that the articles presented as potential risk factors for OC. The formula was constructed based on the product of these odds ratios when each clinical case exhibited these factors. For example, the odds ratio for alcohol consumption is 1.07, so if a patient refers only to alcohol consumption, his or her odds ratio is 1.07, however if the patient has, in addition to alcohol consumption, the smoking habit that is present the value of 4.45, its risk is 1.07 times 4.45, giving a final value of 4.76 ^21^.

After the development of the alpha version, two specialists in stomatology evaluated the algorithm and suggested the addition of risk factors related to sun exposure, whose prevalence ratios were extracted from a case-control study that evaluated risk factors for lip cancer ^22^. Experts also suggested adding risk factors related to oncogenic viruses such as Human Papilloma Virus (HPV), extracting odds ratios of risk factors for oral cancer related to sexual exposure described by Chancellor ^23^.

**Figure 1:**
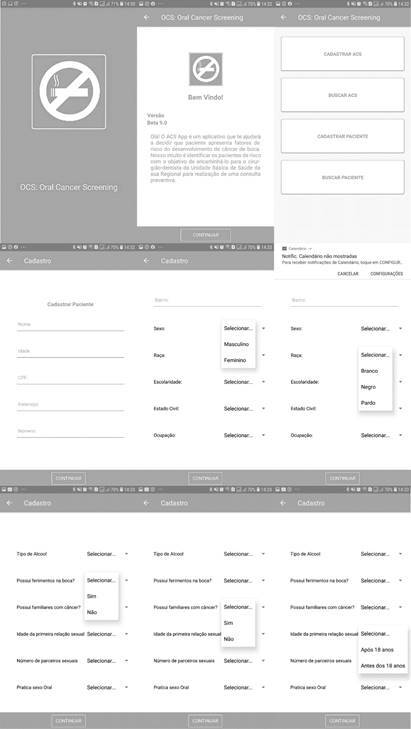
Patient registration screens and entry of clinical data and risk factors for oral cancer from the OCS *App*lication. OCS, Oral Cancer Screening

These odds ratios were included in the algorithm in the same way as the other variables, multiplication, and inserted in the “beta” version of *App*. The entire layout and interface of the *App* were designed (Figure 1) to be easy to understand, easy to visualize, and easy to handle, making it easy to use. The *App* was named the Oral Cancer Scan (OCS).

### Simulated clinical case design for expert validation of the Application

Based on the study by Schoemans et al. ^24^, who observed that the use of an *App* for diagnostic support in graft versus host disease in patients after bone marrow transplantation significantly increases diagnostic hit rates (62%-89%), it is necessary to evaluate the *App* by 40 professionals to obtain a sample that represents 80% power and 95% confidence in the usability of health professionals facing diagnostic support *App*lications.

Based on Andrade et al. ^20^ and Hair et al. ^25^, 10 clinical cases were prepared with basic data on risk factors: ^1^ a 20-year-old female, black, receptionist who drinks fermented alcoholic beverages (beer) twice a week, non-smoker; ^2^ a 67-year-old male retired, brown, pipe smoker for 50 years, daily user of distilled alcoholic beverage (sugarcane liquor) for 40 years; ^3^ a 54-year-old male, brown, bricklayer, non-smoker, who does not consume alcohol and has a lesion on the tongue border for 1 week that prevents him from eating; ^4^ a 60-year-old female, black, retired, former cigarette smoker for 30 years who does not consume alcohol; ^5^ a 57-year-old male, white waiter, non-smoker, former consumer of distilled beverage (vodka) for 20 years; ^6^ a 43-year-old male, black, motorcycle taxi driver, non-smoker, who has been consuming a distilled alcoholic beverage (cachaça) daily for 20 years, presenting a nonpainful wound under the tongue for *App*roximately 1 month; ^7^ a 50-year-old housewife, brown, non-smoker, non-drinker of any alcoholic beverages; ^8^ a 27-year-old male, white, fisherman, a smoker for 10 years, non-drinker of alcoholic beverages; ^9^ a 60-year-old male, former fisherman (retired), non-smoker, no alcoholic drinker, two years of skin cancer history; and ^10^ a 40-year-old female, brown, teacher, cigarette smoker for 20 years, who drinks fermented beverage (wine) more than twice a week.

Next, 40 specialists in the area (stomatology, oral pathology) and related areas (dental care for patients with special needs and oral and maxillofacial surgery) were selected to test the *App* with the 10 cases outlined by the team.

### Usability and acceptability analysis

After the referral of the 10 cases, the professionals selected for the validation process were invited to enter the 8^th^ simulated case in the *App* and then fill out the usability questionnaire System Usability Score (SUS) ^26^. SUS is a questionnaire with 10 items (1: ''I think I would like to use this system often''; 2: ''I find the system unnecessarily complex''; 3: ''I found the system easy to use''; 4: ''I think I would need help from a person with technical knowledge to use the system''; 5: ''I think that the various functions of the system are very well integrated''; 6: ''I think the system has a lot of inconsistency''; 7: ''I imagine people will learn how to use this system quickly''; 8: ''I found the system cumbersome to use''; 9: ''I felt confident using the system''; 10: ''I had to learn a lot of new things before I could use the system'') with five response options arranged in the form of a Likert scale characterized as an easy-to-*App*ly model for assessing the usability of systems ^27^.

Additionally, the professionals were submitted to acceptability evaluation through the Technology Acceptance Model (TAM) questionnaire adapted for the survey inventory proposed by Davis (Questionnaire with four items 1: ''It seems to me to be a useful technology to assess which patient is at risk for oral cancer.''; 2: ''I believe that the standardization through a step-by-step *App*roach proposed by the *App* can help in the identification and referral of patients at risk for oral cancer.''; 3: ''It helped me to better understand the concepts related to risk factors for oral cancer.''; 4: ''You would use the *App* in your routine of home visits/consultations.''; and with 5 response options: Strongly Disagree, Disagree, Indifferent, Agree, Strongly Agree), which allows the quantification of the degree of perceived usefulness by users of a particular *App*lication by sum of scores from four items with five response options arranged on a Likert-type scale ^28^.

To calculate the SUS, 1 was subtracted from the score for the positively (odd) written answers, and 5 was subtracted from the negatively (even) written answers to sum the resulting scores and multiply them by 2.5 to obtain the final score, which can range from 0 to 100 ^29^. For the TAM, the sum of the four responses was multiplied by 5 to obtain the final score, ranging from 0 to 100 ^28^.

### App Interface: OCS

Figure 1 shows the first screen of the beta version of the OCS *App*lication. After clicking on the central icon the user is forwarded to a brief presentation of the *App* objectives; then, clicking continue opens the platform for access and registration of CHAs and patients. On the registration screen of the CHA, it is possible to insert the name, age, Individual Registration (IR), education, and sex of the CHA.

Once registered, the patient can be searched on the previous screen by tracking their IR. On the patient’s registration screen, it is possible to insert the patient’s name, age, IR, address, and residence number. This screen is a continuous screen in which the scrolling mechanism up and down allows the insertion of risk factors to be searched. As shown in the figures, for the item “sex,” male and female options are available. For “race,” the options white, black, and brown are available. In both items, only one answer is allowed, and after the choice is made, the item is selected, and the item selection bar closes so that the user can proceed to fill in the other items (Figure 1).

### Statistical analysis

The SUS and TAM scores are expressed as mean ± standard deviation. They were evaluated by Cronbach’s alpha coefficient and Spearman correlation and analyzed using the Mann-Whitney U test. After categorization (Based on the study by Lewis et al. ^30^ up to 80% vs. >80%), the SUS and TAM scales were associated with other socio-economic variables (Sex, Age, Majored, Professional Training, Higher degree and others) using Pearson’s Fisher’s exact/chi-square tests.

The scores generated by the *App* algorithm mentioned in topic *Development of a risk stratification algorithm for oral cancer* in the validation process were submitted to the receiver operating characteristic (ROC) curve based on the experts’ referral opinion to estimate the cutoff point of the *App* for referral suggestion. All analyses were performed with 95% confidence intervals (CIs) in the Statistical Package for the Social Sciences software for Windows version 20.0, with 95% confidence.

## Results

### Usability and acceptability tests of the App with professionals specialized in areas related to oral diagnosis

Forty professionals responded to the OCS usability and acceptability tests. The positive items of the SUS showed a high internal validity index (Cronbach’s α=0.869), and the negative items showed a median internal validity index (Cronbach’s α = 0.629). The TAM also showed adequate internal validity (Cronbach's α=0.729).

Among the positive items of the SUS, the highest mean scores in descending order were noted in item 3 (4.65±0.48), item 7 (4.45±0.55), item 5 (4.35±0.58), item 9 (4.33±0.62), and item 1 (4.23±0.80) (p<0.001), respectively. Of the negative items of the SUS, the lowest mean scores described in ascending order were noted in item 8 (1.48±0.51), item 10 (1.60±0.55), item 4 (1.65±0.80), item 6 (1.70±0.52), and item 2 (1.73±0.78), respectively; however, there were no significant differences among these (p=0.169). Of the TAM items, the highest mean scores were described in item 2 (4.53±0.51), item 1 (4.45±0.50), item 4 (4.33±0.69), and item 3 (3.65±1.03), respectively (p<0.001).

**Figure 2:**
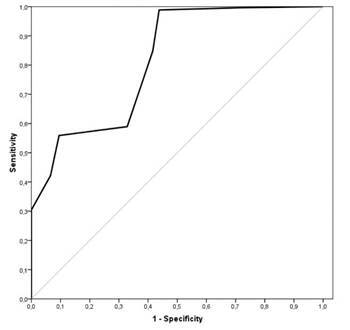
ROC curve for estimating the suggestive cutoff point for referring patients for preventive consultations at the primary care unit for being at high risk of developing oral cancer. ROC, receiver operating characteristic

The average score for usability was 84.63±10.66, ranging from 70 to 100, and that for acceptability was 84.75±10.62, ranging from 70 to 100. There was no significant difference between acceptability and usability (p=0.698), and they correlated significantly with each other (p<0.001, r=0.647) (Figure 2).

### Indicators of usability and acceptability of the App with professionals specialized in areas related to oral diagnosis

Most users rated the OCS *App* as having a high level of usability (>80) (n=21, 52.5%) and acceptability (>80) (n=26, 65.0%). Most professionals interviewed were male, aged > 35 years, and had majored less than 10 years ago. The most mentioned professional background was stomatology, followed by dentistry for patients with special needs, with the doctorate as the maximum degree of being the most mentioned. Most professionals had their last post-graduation in areas related to oral diagnosis < 3 years ago and had another specialization. None of these variables interfered with usability; however, professionals trained in oral pathology had low acceptability (TAM>80) (p=0.002), while professionals specialized in Oral and Maxillofacial Surgery and Traumatology mentioned high acceptability (TAM>80) (p=0.034) (Table 1). 

Most participating professionals work in the teaching and private sectors and perform biopsies, including potentially malignant and malignant lesions of the oral cavity, with a frequency of more than three biopsies per month. None of these variables interfered with acceptability, but professionals working in the teaching sector rated higher usability (>80) (p=0.011) (Table 2).

Smoking (mouth) and sun exposure (lip), alcohol consumption, and human papillomavirus infection were risk factors for mouth cancer in 95% of the sample. Three-quarters of the professionals believe that unprotected oral sex, advanced age, and immunosuppression are risk factors for oral cancer. Almost all evaluated professionals also mentioned counseling about risk factors for oral cancer (95.0%). 

None of these factors was associated with changes in usability. However, the professionals who mentioned that unprotected oral sex is a risk factor for oral cancer showed lower acceptability of the *App* (p=0.007), and the professionals who provided guidance on risk factors for oral cancer showed higher acceptability (p=0.048) (Table 2).

Most users evaluated used iOS (n=21, 52.5%), and the prevalence of high usability in Android (n=9, 47.4%) and iOS (n=12, 57.1%) users did not significantly differ (p=0.563). Acceptability also did not significantly differ between Android (n=12, 63.2%) and iOS (n=16, 66.7%) users (p=0.816). 

**Table 1:** Influence of sociodemographic factors on the usability and acceptability of the OCS app by professionals with expertise in oral diagnosis.

		SUS			TAM		
	Total	Up to 80	>80	p-Value	Up to 80	>80	p-Value
Sex							
Female	15 (37.5%)	7 (36.8%)	8 (38.1%)	0.935	5 (35.7%)	10 (38.5%)	0.864
Male	25 (62.5%)	12 (63.2%)	13 (61.9%)		9 (64.3%)	16 (61.5%)	
Age							
Up to 35	19 (47.5%)	9 (47.4%)	10 (47.6%)	0.987	9 (64.3%)	10 (38.5%)	0.119
> 35	21 (52.5%)	10 (52.6%)	11 (52.4%)		5 (35.7%)	16 (61.5%)	
Majored							
<10 years	21 (52.5%)	8 (42.1%)	13 (61.9%)	0.210	8 (57.1%)	13 (50.0%)	0.666
10+	19 (47.5%)	11 (57.9%)	8 (38.1%)		6 (42.9%)	13 (50.0%)	
Professional Training							
Stomatology	18 (45.0%)	9 (47.4%)	9 (42.9%)	0.775	6 (42.9%)	12 (46.2%)	0.842
SNP Dentistry	15 (37.5%)	9 (47.4%)	6 (28.6%)	0.220	7 (50.0%)	8 (30.8%)	0.231
Oral Pathology	9 (22.5%)	4 (21.1%)	5 (23.8%)	0.835	7 (50.0%)*	2 (7.7%)	*0.002*
OMST	11 (27.5%)	4 (21.1%)	7 (33.3%)	0.385	1 (7.1%)	10 (38.5%)*	*0.034*
Higher degree							
Residency	2 (5.0%)	2 (10.5%)	0 (0.0%)	0.101	1 (7.1%)	1 (3.8%)	0.371
Specialization	4 (10.0%)	3 (15.8%)	1 (4.8%)		1 (7.1%)	3 (11.5%)	
Masters	15 (37.5%)	4 (21.1%)	11 (52.4%)		3 (21.4%)	12 (46.2%)	
Doctorate	19 (47.5%)	10 (52.6%)	9 (42.9%)		9 (64.3%)	10 (38.5%)	
Time of last graduate degree in a related field							
Up to 3 years	28 (70.0%)	11 (57.9%)	17 (81.0%)	0.112	10 (71.4%)	18 (69.2%)	0.885
>3 years	12 (30.0%)	8 (42.1%)	4 (19.0%)		4 (28.6%)	8 (30.8%)	
Has another specialization							
No	16 (40.0%)	6 (31.6%)	10 (47.6%)	0.301	5 (35.7%)	11 (42.3%)	0.685
Yes	24 (60.0%)	13 (68.4%)	11 (52.4%)		9 (64.3%)	15 (57.7%)	

* p<0.05, Fisher's exact test or Pearson's chi-square test (n, %). SNP = Special Needs Patients; OMST = Oral and Maxillofacial Surgery and Traumatology.

### Determination of cutoff scores for the suggestion of referral of at-risk patients by the CHA for preventive consultation in the primary care unit

According to the algorithm suggested by the *App* with the information available in each case, they presented the score described below.

Based on these cases and opinion, the ROC curve showed a significant predictive value (p<0.001), with an area under the curve of 0.815±0.022 (95% CI=0.771-0.859) (Figure 2). Two cutoff points were suggested based on the ROC curve to achieve the best accuracy values. The cutoff point suggesting the largest area under the curve was 10 points.

The cutoff point suggested by the *App* of 10 points obtained a sensitivity of 84.8%, specificity of 58.4%, positive predictive value of 79.6%, negative predictive value of 66.7%, accuracy of 75.8%, and likelihood ratio of 7.82 (95% CI=4.85-12.6). The k*App*a index between the *App* and professionals opinions was k*App*a = 0.445±0.48.

**Table 2:** Influence of professional experience and knowledge about risk factors for oral cancer on the usability and acceptability of the OCS app by professionals with expertise in oral diagnosis.

		SUS			TAM		
	Total	Up to 80	>80	p-Value	Up to 80	>80	p-Value
Professional practice in oral diagnosis							
Teaching	32 (80.0%)	12 (63.2%)	20 (95.2%)*	*0.011*	10 (71.4%)	22 (84.6%)	0.320
Private sector	27 (67.5%)	15 (78.9%)	12 (57.1%)	0.141	10 (71.4%)	17 (65.4%)	0.697
Public sector	14 (35.0%)	7 (36.8%)	7 (33.3%)	0.816	5 (35.7%)	9 (34.6%)	0.945
Has performed biopsies							
No	11 (27.5%)	4 (21.1%)	7 (33.3%)	0.385	4 (28.6%)	7 (26.9%)	0.911
Yes	29 (72.5%)	15 (78.9%)	14 (66.7%)		10 (71.4%)	19 (73.1%)	
Monthly biopsies							
Up to 3	10 (34.5%)	7 (46.7%)	3 (21.4%)	0.153	5 (50.0%)	5 (26.3%)	0.202
>3	19 (65.5%)	8 (53.3%)	11 (78.6%)		5 (50.0%)	14 (73.7%)	
Do you do biopsies of malignant lesions?							
No	10 (25.0%)	3 (15.8%)	7 (33.3%)	0.201	4 (28.6%)	6 (23.1%)	0.702
Yes	30 (75.0%)	16 (84.2%)	14 (66.7%)		10 (71.4%)	20 (76.9%)	
Do you do biopsies of potentially malignant lesions?							
No	10 (25.0%)	3 (15.8%)	7 (33.3%)	0.201	4 (28.6%)	6 (23.1%)	0.702
Yes	30 (75.0%)	16 (84.2%)	14 (66.7%)		10 (71.4%)	20 (76.9%)	
Risk factors for oral cancer							
Smoke	40 (100.0%)	19 (100.0%)	21 (100.0%)	1.000	14 (100.0%)	26 (100.0%)	1.000
Alcohol	38 (95.0%)	18 (94.7%)	20 (95.2%)	0.942	14 (100.0%)	24 (92.3%)	0.287
HPV infection	38 (95.0%)	19 (100.0%)	19 (90.5%)	0.168	14 (100.0%)	24 (92.3%)	0.287
Sun exposure (lips)	40 (100.0%)	19 (100.0%)	21 (100.0%)	1.000	14 (100.0%)	26 (100.0%)	1.000
Unprotected oral sex	30 (75.0%)	14 (73.7%)	16 (76.2%)	0.855	14 (100.0%)*	16 (61.5%)	*0.007*
Advanced age	25 (62.5%)	9 (47.4%)	16 (76.2%)	0.060	10 (71.4%)	15 (57.7%)	0.392
Drug use	9 (22.5%)	5 (26.3%)	4 (19.0%)	0.583	2 (14.3%)	7 (26.9%)	0.361
Poorly fitted prostheses	9 (22.5%)	5 (26.3%)	4 (19.0%)	0.583	2 (14.3%)	7 (26.9%)	0.361
Unprotected sex	10 (25.0%)	6 (31.6%)	4 (19.0%)	0.361	4 (28.6%)	6 (23.1%)	0.702
Low socioeconomic status	15 (37.5%)	6 (31.6%)	9 (42.9%)	0.462	7 (50.0%)	8 (30.8%)	0.231
Immunosuppression	25 (62.5%)	10 (52.6%)	15 (71.4%)	0.220	10 (71.4%)	15 (57.7%)	0.392
Poor oral hygiene	12 (30.0%)	4 (21.1%)	8 (38.1%)	0.240	2 (14.3%)	10 (38.5%)	0.112
Advises on risk factors for oral cancer							
No	2 (5.0%)	2 (10.5%)	0 (0.0%)	0.127	2 (14.3%)*	0 (0.0%)	*0.048*
Yes	38 (95.0%)	17 (89.5%)	21 (100.0%)		12 (85.7%)	26 (100.0%)*	

* p<0.05, Fisher's exact test or Pearson's chi-square test (n, %). HPV = human papilloma virus.

The second cutoff point suggested by the *App* was >20 points. This cutoff point obtained a lower sensitivity (71.9%) but a slightly higher specificity (62.8%), equivalent positive predictive value (78.8%), lower negative predictive value (53.8%), slightly lower accuracy (68.8%), and lower likelihood ratio of 4.30 (95% CI = 2.78-6.68), although without significant difference with the previous cutoff point. The k*App*a index between the *App* and professionals’ opinions was 0.333±0.48.

Thus, we opted for a cutoff point >10 as moderately suggestive for referral to preventive consultation with the Basic Health Unit dental surgeon and >20 as strongly suggestive. After validation, the *App* was registered as a computer program at the National Institute of Intellectual Property (BR512020002942).

**Table 3:** Result of the products of the risk factor scores of the ten clinical cases outlined for simulation and development of the formula/algorithm for the application to suggest to the CHA the referral of patients at risk to the primary care unit.

Case	Risk Factors								Score
1	Brown/Black	Alcohol	Alcohol 2x a week						2,46
	x1,33	x1,07	x1,73						
2	Brown	Smoker	Pipe smoking	Smoking >20 yrs	Alcohol	Alcohol >2x a week	Distilled beverage	Drink >20 yrs	26.632,20
	x1,33	x4,45	x5,06	x7,24	x1,07	x5,54	x5,87	x3,53	
3	Brown	Sun exposure while work	Oral lesion						6477,1
	x1,33	x4,87	x1000						
4	Black	Former smoker	Cigarette smoking	Smoking >20 yrs					14,48
	x1,33	x1,38	x1,09	x7,24					
5	Former drinker	Distilled beverage	Drink >20 yrs						56,57
	x2,73	x5,87	x3,53						
6	Black	Sun exposure during work	Alcohol	Distilled beverage	Alcohol>2x a week	Drink	Oral lesion		795585,7
	x1,33	x4,87	x1,07	x5,87	x5,54	x3,53	x1000		
7	Brown								1,33
	x1,33								
8	Sun exposure during work	Smoker							21,67
	x4,87	x4,45							
9	Sun exposure during work								4,87
	x4,87								
10	Brown	Smoker	Cigarette smoking	Smoking >20 yrs	Alcohol	Alcohol >2x a week			276,87
	x1,33	x4,45	x1,09	x7,24	x1,07	x5,54			

**Table 4:** Accuracy of cutoff points suggested by ROC curve for referral of simulated clinical cases for preventive consultation in the primary care unit.

	Referral (expert opinion)							
	No	Yes	S	E	PPV	NPV	A	LR (95%CI)
Referral (App score > 10)								
No	80	40	84.8%	58.4%	79.6%	66.7%	75.8%	7.82 (4.85 - 12.6)
Yes	57	223						
Referral (App score > 20)								
No	86	74	71.9%	62.8%	78.8%	53.8%	68.8%	4.30 (2.78 - 6.68)
Yes	51	189						

S = Sensitivity; E = specificity; PPV = positive predictive value; NPV = negative predictive value; A = accuracy; LR = likelihood ratio; 95%CI = 95% confidence interval of the LR.

## Discussion

A screening program for oral cancer and potentially malignant diseases should be able to detect these diseases before symptoms *App*ear. It is vital that risk factors are identified and, when possible, controlled ^29^ ^,^ ^31^. The study seeks screening focused on risk factors to monitor these users and greater effectiveness in detecting early lesions.

Screening programs are less costly and can detect cancerous or potentially cancerous lesions at early stages, allowing for proper management and contributing to improved treatment outcomes and survival rates ^32^. Moreover, screening programs have the inherent benefit of educating the public, increasing awareness of the signs and symptoms of cancer among the general population ^5^ ^,^ ^32^. However, screening programs occur occasionally in specific areas. The advantage of the *App* is that it can be used routinely.

Webster et al. ^33^ demonstrated the importance of performing asymptomatic screening outside of the dental surgeon’s place of practice. Therefore, public health educators and strategists are urged to increase awareness of oral cancer and the need for opportunistic screening. 

Issues related to the usefulness/purpose and ease of use of a mobile device in health are very relevant because these factors directly influence the success or barriers to using this device by health professionals ^34^. In the survey, we observed a satisfactory level of usability and acceptability, which positively implies a greater possibility of the *App* being used habitually by professionals. The positive evaluation of usability highlights the user’s understanding of the effectiveness and efficiency of the *App*lication, demonstrating their satisfaction ^35^. Furthermore, our study obtained great acceptability; therefore, we expect good results related to the benefits of its use, as was seen in the systematic review with a meta-analysis conducted by Choi in 2020, showing 70% acceptance and satisfaction in the use of *App*lications for exercise monitoring, medication adherence, and heart rate monitoring ^36^ Therefore, this acceptance is related to users’ level of engagement with the tool and is important for increasing the use of these platforms and formulating new strategies to sustain users’ interest and usability and facilitate their use ^36^. 

Our results during the *App* assessment through the scales showed a low intensity of negative items on the SUS scale (example: I find the system unnecessarily complex…), which are important data for the improvement of the *App* in future updates that aim to improve the usability and *App*licability of the system through the thin user's view, justifying the high acceptability values ​​found ^29^. Acceptance of digital solutions and innovative medical technologies by patients and professionals depends on understanding their anxieties and feelings of insecurity. Developing a broad user community to implement e-Health successfully requires long training, capacity building, and enhanced affinity with the technology ^37^.

Farias et al. ^38^ reported that Oral and Maxillofacial Surgery and Traumatology is the specialty that most acts in the care of patients with cancer. The present study observed that professionals in this specialty obtained the best SUS/TAM results compared to other specialists, thus demonstrating greater acceptability of the *App*lication. In contrast, oral pathology specialists are inserted in the laboratory side of the diagnostic process. 

The low adherence of pathologists is a reflection of the distance from their experience in the disease process in a separate way and without contact with the clinician and interpersonal connection with the patient, so that there is difficulty in developing an empathy with him. In a study by Pérez-De-Oliveira et al. ^39^ it was possible to observe the inherent difficulties of the profession for laboratory pathologists regarding the establishment of this connection with the patient, unlike professionals who have a strong clinical dynamic. Therefore, these factors may be directly interconnected with the low adherence of these professionals to the *App*.

The *App*lication *App* showed high sensitivity (84.8%) and moderate specificity (58.4%) caused by a high proportion of false positives and resulting in low k*App*a concordance rates. Previous studies such as that of Patz et al. ^40^, demonstrate that the cost-effectiveness of false positives is something positive for cancer screening, as it increases the proportion of early diagnosis of the disease. The *App* developed in this study aims to make a screening system, so this becomes tolerable, given that the objective is not to diagnose: those patients who are positive according to the *App* will be referred to a basic health unit, where a trained professional will evaluate them and the diagnosis will be defined. Thus, low specificity becomes a favorable limitation to the cancer screening system and is common on the study that evaluated screening tests ^41^ that have in identifying borderline cases. A screening test should prioritize sensitivity to identify the largest possible number of individuals who need healthcare ^41^. However, it may burden the health service over time, which can be minimized by defining more specific risk populations ^33^.

The validation phase of the *App* was conducted based on research with professionals with expertise in the oral diagnosis and SUS and TAM tests, thus presenting a limitation because the practical use will be made through CHAs in primary care units. Therefore, future studies including these professionals are necessary to evaluate the acceptability and usability of the *App*. Considering the evaluated professionals and the limitations of this study, such low specificity that is tolerable in cancer screening systems due high proportions of early diagnosis ^40^, the results showed that the *App* will increase the early diagnosis of oral cancer and reduce healthcare expenses for the treatment of cancers discovered in advanced stages.

## Conclusion

Our team has developed and validated with professionals in the field of oral diagnosis OCS, an *App* created to facilitate the identification of risk factors for oral cancer and aid early diagnosis, either in screening programs or during the dentists’ daily primary care practice or CHAs.
